# Foot-and-mouth disease virus non-structural protein 3A inhibits the interferon-β signaling pathway

**DOI:** 10.1038/srep21888

**Published:** 2016-02-17

**Authors:** Dan Li, Caoqi Lei, Zhisheng Xu, Fan Yang, Huanan Liu, Zixiang Zhu, Shu Li, Xiangtao Liu, Hongbing Shu, Haixue Zheng

**Affiliations:** 1 State Key Laboratory of Veterinary Etiological Biology, National Foot and Mouth Diseases Reference Laboratory, Lanzhou Veterinary Research Institute, Chinese Academy of Agricultural Sciences, Lanzhou 730046, China; 2 Collaborative Innovation Center for Viral Immunology, Medical Research Institute, Wuhan University, Wuhan 430072, China

## Abstract

Foot-and-mouth disease virus (FMDV) is the etiological agent of FMD, which affects cloven-hoofed animals. The pathophysiology of FMDV has not been fully understood and the evasion of host innate immune system is still unclear. Here, the FMDV non-structural protein 3A was identified as a negative regulator of virus-triggered IFN-β signaling pathway. Overexpression of the FMDV 3A inhibited Sendai virus-triggered activation of IRF3 and the expressions of RIG-I/MDA5. Transient transfection and co-immunoprecipitation experiments suggested that FMDV 3A interacts with RIG-I, MDA5 and VISA, which is dependent on the N-terminal 51 amino acids of 3A. Furthermore, 3A also inhibited the expressions of RIG-I, MDA5, and VISA by disrupting their mRNA levels. These results demonstrated that 3A inhibits the RLR-mediated IFN-β induction and uncovered a novel mechanism by which the FMDV 3A protein evades the host innate immune system.

Foot-and-mouth disease virus (FMDV) is the etiological agent of foot-and-mouth disease (FMD) that is highly contagious for affect domestic and wild cloven-hoofed animals worldwide. There are seven known serotypes of FMDV (A, O, Asia1, C, SAT1, SAT2, and SAT3), which consists of numerous subtypes^1^,^2^. The FMDV genome is an 8.5 kb, positive sense, single-stranded RNA molecule containing one open reading frame (ORF). The ORF encodes a single polyprotein that is post-translationally processed into four structural proteins (VP1–4) and eight non-structural proteins (L^pro^, 2A, 2B, 2C, 3A, 3B, 3C^pro^, and 3D)^3^. Similar to most viruses, FMDV has evolved to inhibit or evade the innate immune system. Innate immunity plays an important role in defending the host from invading pathogens. Type I interferons (IFNs), such as IFN-α and IFN-β, are recognized as an essential component of the innate immune response, especially against viral infections. The recognition of pathogen-associated molecular patterns (PAMPs) is conducted by a series of pattern recognition receptors, including toll-like receptor (TLRs) and retinoic acid-inducible gene-I (RIG-I)-like receptors (RLRs)^4^,^5^,^6^. TLRs are mostly expressed by macrophages, dendritic cells, and other immune cells, where they detect PAMPs at the cell surface or within endosomes^7^. In contrast, RIG-I and melanoma differentiation-associated gene 5 (MDA-5) are localized in the cytosol and mainly function in detecting RNA virus. RIG-I like receptors (RLRs) compose two copies of a caspase recruitment domains (CARD) at the N terminus, a regulatory/repression domain at the C terminus, and a central DExD/H box ATPase/helicase. Following activation after viral infection, the CARDs of RIG-I or MDA-5 are released and interact with the CARD motif of VISA, which subsequently lead to the activations of IFN regulatory factor 3 (IRF3) and nuclear factor-κB (NF-κB)^8^,^9^,^10^. Upon activation, IRF3 and NF-κB translocate to the nucleus and collaborate in inducing expression of a series of genes, such as type I IFNs, interferon-stimulated genes (ISG) and inflammatory cytokines^11^,^12^.

In order to replicate and spread in the hosts, many pathogens subvert the host cellular defense machinery to combat innate immune system, such as FMDV has evolved to actively suppress the production of host interferon *in vitro*^13^,^14^,^15^. Furthermore, FMDV or some proteins of FMDV could antagonize the host innate response in mammalian cells by preventing the translational^16^ or transcriptional level of host proteins^17^. Such as, the FMDV leader proteinase (L^pro)^ plays a key role in preventing IFN-α/β protein synthesis, by reducing the level of immediate-early induction of IFN-β mRNA and IFN stimulated gene products^16^, degrading p65/RelA^18^ or IRF3/7^19^, cleaving ubiquitin moieties from critical signaling proteins of the type I IFN signaling pathway, and/or abrogating the deubiquitinating activity of enzymes^20^.

In addition, FMDV 3C^pro^ inhibits the virus-triggered expression of IFN-α and -β expression by cleaving NEMO (NF-κB essential modulator)^21^. It was recently reported that 3C^pro^ antagonizes the IFN signaling pathway by blocking the nuclear translocation of STAT1 and STAT2^17^. FMDV VP1 suppresses type I IFN induction by interacting with sorcin, which appears to regulate the cellular response to viral infections^22^. It remains unclear whether other FMDV nonstructural proteins are involved in inhibition of type I IFN signaling. In the present study, the results demonstrated that FMDV 3A inhibited virus-triggered the IFN-β signaling pathway. It also inhibited the expression of RIG-I, MDA5 and VISA proteins by disrupting their mRNA levels. It uncovered the complicated mechanisms underlying the evasion of the host immune response by FMDV.

## Results

### FMDV 3A is a negative regulator of virus-triggered IFN-β expression

**Figure 1:**
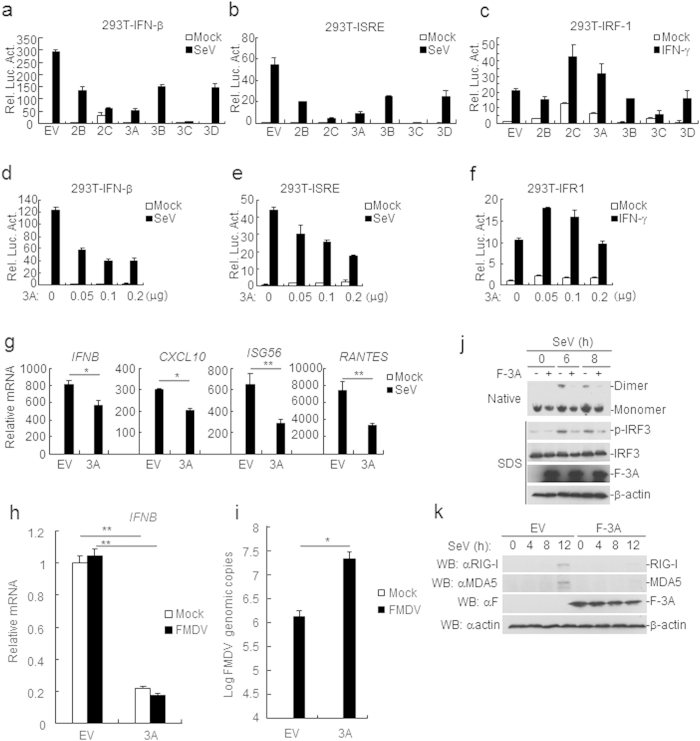
FMDV 3A protein inhibits the SeV-induced IFN-β signaling pathway. (**a,b**) Effects of overexpression of FMDV nonstructural proteins on SeV-triggered IFN-β promoter or IRSE activation. HEK293T cells (5 × 10^4^) were transfected with the IFN-β or ISRE reporter (0.1 μg), 10 ng of pRL-TK (as an internal control) and the indicated expression plasmids (0.1 μg) for 24 h. Cells were infected or uninfected with SeV for 12 h before luciferase assays were performed. (**c**) Effects of overexpression of FMDV proteins on IFNγ-triggered IRF1 activation. The experiments were similarly performed as in a. (**d–f**) FMDV 3A inhibits SeV- or IFNγ-induced activation of IFN-β, ISRE, and IRF1 promoter in a dose-dependent manner. The experiments were similarly performed as in a. (**g**) FMDV 3A inhibits SeV-induced transcription of endogenous *Ifnb*, *Cxcl-10*, *Isg56* and *Rantes* genes. HEK293T cells were transfected with 4 μg FMDV 3A or empty vector (EV) for 24 h followed by SeV infection for 12 h before a relative quantitative RT-PCR experiments were performed. (**h**) 3A inhibited the transcriptional level of the endogenous IFN-β mRNA in PK-15 cells. PK-15 (2 × 10^5^) cells were transfected with 4 μg pMSCV-3A plasmid using lipofectamine 2000 for 12 h, subsequently treated with puromycin (1 mg/ml) for 24 h. Cells were then infected with the type O FMDV (moi: 0.1) for 6 h. IFN-β mRNA abundance in PK-15 cells was assessed using a relative quantitative RT-PCR. (**i**) Increased FMDV genome copies in 3A-transfected PK-15 cells. The experiments were similarly performed as in i. FMDV genome copies were assessed using a quantitative RT-PCR assay. (**j,k**) FMDV 3A inhibits phosphorylation and dimerization of IRF3 and the expression of RIG-I and MDA5 after SeV stimulation. HEK293T (2 × 10^5^) cells were transfected with 4 μg the FMDV 3A plasmid or EV for 24 h. Cells were infected with SeV at various time points and then harvested for analysis by western blotting. Values are presented as mean ± SD of three independent experiments. Rel. Luc. Act., relative luciferase activity.

To address the potential roles of FMDV nonstructural proteins in regulating cellular antiviral response, the effects of FMDV nonstructural proteins on virus-triggered activation of IFN-β were examined by reporter assays. The data showed that 3A could markedly inhibited Sendai virus (SeV)-triggered activations of the IFN-β promoters (Fig. 1a) and ISRE (an IRF3-binding motif) (Fig. 1b), but not affect IFNγ-induced activation of IRF1 promoter (Fig. 1c). Overexpression of FMDV 3A significantly inhibited SeV-triggered activation of the IFN-β promoter and ISRE in a dose-dependent manner in human embryonic kidney 293T (HEK293T) cells (Fig. 1d,e), while IFNγ-induced activation of IRF1 promoter was not affected (Fig. 1f). In real-time PCR experiments, it was observed that SeV-triggered transcriptions of the *Ifnb1*, *Cxcl-10*, *Isg56* and *Rantes* genes were decreased in FMDV 3A-transfected cells in comparison with the control transfected cells (Fig. 1g). To investigate the relevance of the activation of the IFN-β signaling pathway in FMDV replication, the infectious progeny virus of the type O FMDV in 3A-transfected PK-15 cells was examined. The transcriptional level of IFN-β was reduced in the 3A-transfected PK-15 cells (Fig. 1h), indicating that the activation of the IFN-β signaling pathway was inhibited by FMDV 3A. Consistent with the decrease of IFN-β mRNA level, the FMDV genome copies were increased in the 3A-transfected PK-15 cells when compared with non-transfected cells (Fig. 1i). Furthermore, it was found that overexpression of the FMDV 3A inhibited IRF3 phosphorylation and dimerization, which are hallmarks for IRF3 activation (Fig. 1j), in addition, overexpression of FMDV 3A decreased the expressions of RIG-I/MDA5 after SeV infection (Fig. 1k). Collectively, these results suggest that FMDV 3A is a negatively regulator of virus-triggered IFN-β induction.

### FMDV 3A negatively regulates virus-triggered signaling at or upstream of the VISA level

**Figure 2:**
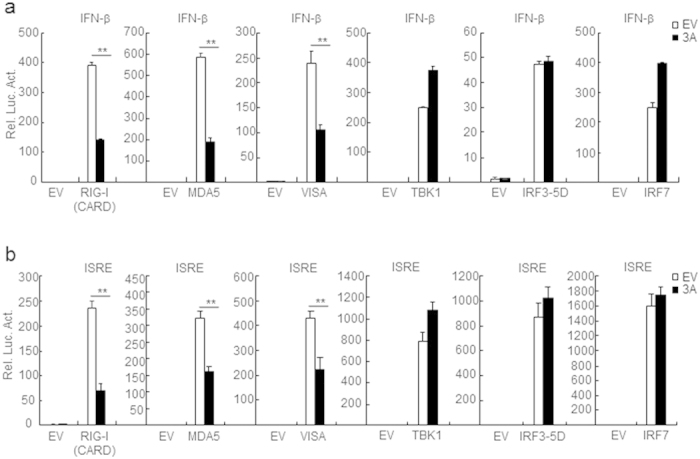
FMDV 3A protein targets at or upstream of VISA. (**a,b**) Effects of FMDV 3A on IFN-β or ISRE activation with various signaling components. HEK293T cells (5 × 10^4^) were transfected with IFN-β or ISRE reporter (100 ng), 10 ng of pRL-TK (as an internal control), FMDV 3A expression plasmids (100 ng) and indicated proteins (100 ng each). Luciferase assays were performed 24 h after transfection. Values are presented as the mean ± SD from three independent experiments. Rel. Luc. Act., relative luciferase activity.

There are various components involved in IFN-β signaling pathways. In order to determine the molecular mechanisms of FMDV 3A in regulating RLR-mediated antiviral pathways, we co-transfected FMDV 3A with RIG-I (CARD)/MDA5 or their downstream signaling proteins and examined the activations by dual-luciferase assays. Overexpression of FMDV 3A inhibited activation of the IFN-β promoter induced by VISA as well as its upstream components RIG-I (CARD) and MDA5, but did not affect IFN-β promoter activation induced by VISA downstream components TBK1, IRF3–5D and IRF7 (Fig. 2a). Consistently, overexpression of FMDV 3A inhibited activation of the ISRE induced by RIG-I (CARD), MDA5, and VISA, but not TBK1, IRF3-5D and IRF7 (Fig. 2b). These data suggest that FMDV 3A targets at or upstream of VISA to regulate RLR-mediated signaling pathway.

### FMDV 3A interacts with RIG-I, MDA5, and VISA

**Figure 3:**
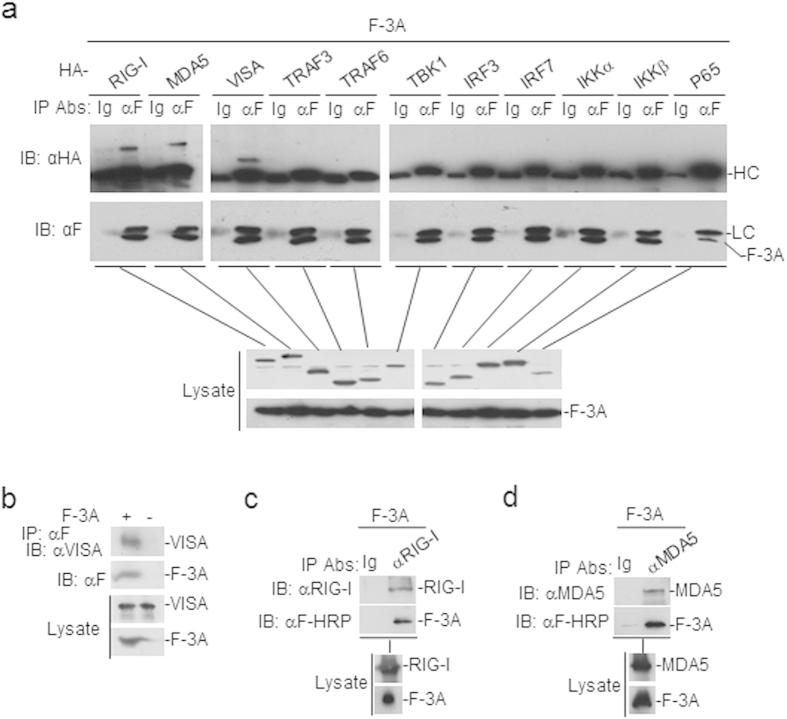
FMDV 3A interacts with RIG-I, MDA5, and VISA. (**a**) FMDV 3A interacts with the overexpression of RIG-I, MDA5 and VISA proteins. The HEK293T cells (2 × 10^6^) were cotransfected with 4 μg of FMDV Flag-3A plasmid and the indicated HA-tagged plasmids (5 μg each). Coimmunoprecipitations were performed with anti-Flag (F) or control IgG (lg) antibodies. Immunoblot analysis was performed with anti-hemagglutinin (HA) (upper panels) antibody. Expression levels of the proteins were analyzed by immunoblot analysis of the lysates with anti-HA and anti-Flag (lower panels). HC: heavy chain; LC: light chain. (**b–d**) FMDV 3A is associated with endogenous RIG-I, MDA5 and VISA. The HEK293T cells (1 × 10^7^) were cotransfected with 20 μg of FMDV Flag-3A plasmid for 24 hour. Coimmunoprecipitations were then performed with anti-Flag (F), anti-RIG-I, anti-MDA5 or control IgG (lg) antibodies. Immunoblot analysis was performed with anti-VISA, anti-RIG-I or anti-MDA5 (upper panels) antibodies. Expression levels of the proteins were analyzed by immunoblot analysis of the lysates with anti-VISA, anti-RIG-I, anti-MDA5, and anti-Flag-HRP (F-HRP) (lower panels). HA-: HA tag.

To further evaluate the complicated regulatory mechanisms of FMDV 3A in virus-triggered signaling pathway, we examined the ability of FMDV 3A to associate with RIG-I, MDA5, and VISA which were shown to be inhibited in report assays. In transient transfection and co-immunoprecipitations experiments, FMDV 3A was found to specifically interacts with VISA and its upstream sensor RIG-I and MDA5, but not with TRAF3, TRAF6, TBK1, IRF3, IRF7, IKKα, IKKβ or P65 (Fig. 3a). Furthermore, to determine whether FMDV 3A interacts with RIG-I/MDA5/VISA in physiological conditions, we did co-immunoprecipitation experiments with antibodies recognizing endogenous RIG-I, MDA5, and VISA. The results showed FDMV 3A could interact with endogenous RIG-I, MDA5, and VISA (Fig. 3b–d). Taken together, these results suggest that FMDV 3A interacts with RIG-I, MDA5 and VISA.

### Domain mapping of the FMDV 3A interaction

**Figure 4:**
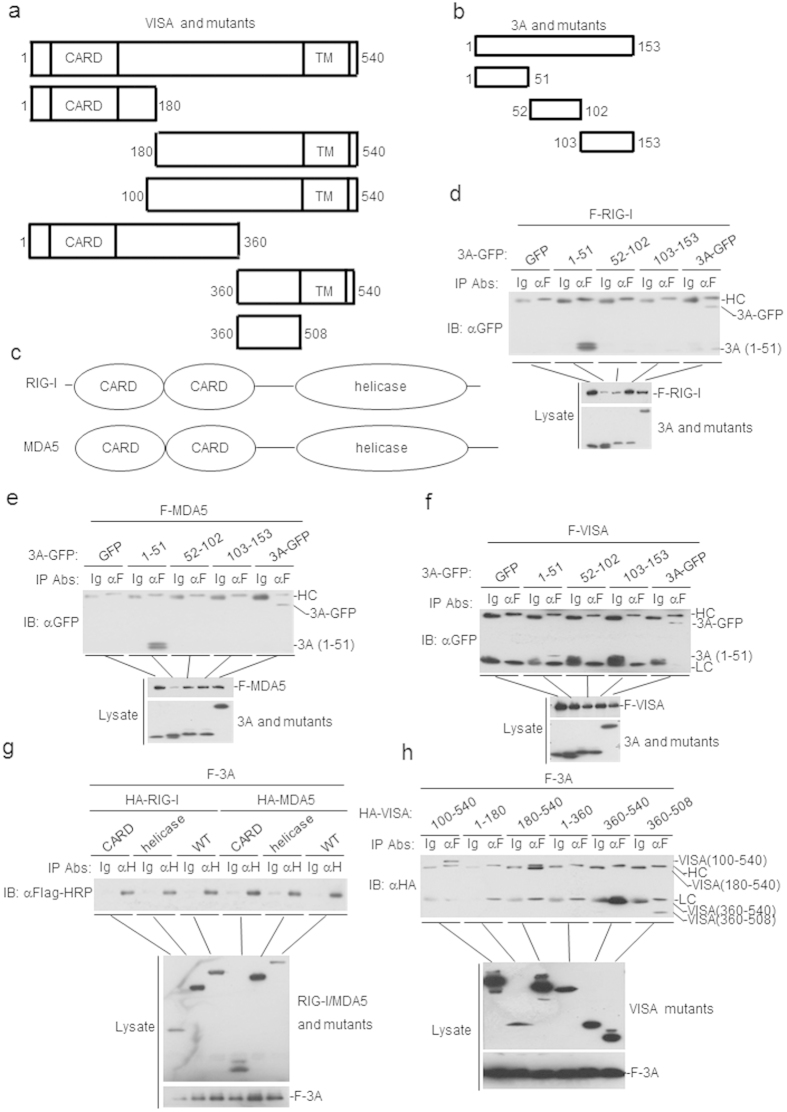
FMDV 3A and its mutants interact with RIG-I, MDA5, VISA and their mutants. (**a–c**) A schematic diagram of full-length and truncated FMDV 3A, RIG-I, MDA5 and VISA and their mutants. (**d–f**) Amino acids 1–51 of 3A are required for its interaction with RIG-I, MDA5, and VISA. The HEK293T cells (2 × 10^6^) were cotransfected with 4 μg of FMDV GFP-3A plasmid or its mutants and the plasmids expressing Flag-RIG-I (F-RIG-I), Flag-MDA5 (F-MDA5) or Flag-VISA (F-VISA) (5 μg each). Coimmunoprecipitations were performed with anti-Flag (F) or control IgG (lg) antibodies. Immunoblot analysis was performed with anti-green fluorescent protein (GFP) (upper panels). Expression levels of the proteins were analyzed by immunoblot analysis of the lysates with antibodies against anti-Flag and anti-GFP (lower panels). (**g,h**) Amino acids 360–540 of VISA, and the CARD and helicase domains of RIG-I/MDA5 interacts with 3A. The HEK293T cells (2 × 10^6^) were co-transfected with full-length or truncated HA-RIG-I (10 μg), HA-MDA5 (10 μg) or HA-VISA (4 μg) and Flag-3A (4 μg) (F-3A). Coimmunoprecipitations were performed with anti-HA (H), anti-Flag (F) or control IgG (lg) antibodies. Immunoblot analysis was performed with antibodies against anti-Flag-HRP or anti-HA. Expression levels of the proteins were analyzed by immunoblot analysis of the lysates with anti-HA and anti-Flag (lower panels). HC: heavy chain; LC: light chain.

**Figure 5:**
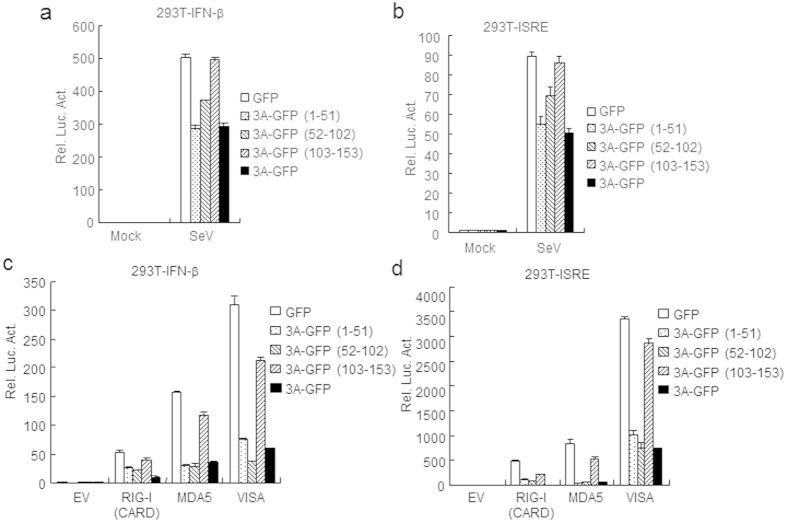
Amino acids 1–102 of FMDV 3A inhibits IFN-β signaling pathway by reporter assay. (**a,b**) The key domain of FMDV 3A inhibits SeV-induced IFN-β signaling pathway. HEK293T (5 × 10^4^) cells were transfected with the IFN-β reporter or ISRE (100 ng), the indicated expression plasmids (100 ng), and 10 ng of pRL-TK (as an internal control). Twenty hours after transfection, cells were infected with SeV or left uninfected for 12 h before luciferase assays were performed. (**c,d**) The key domain of FMDV 3A inhibits the IFN-β signaling pathway. HEK293T (5 × 10^4^) cells were transfected with the IFN-β reporter or ISRE (100 ng), the indicated expression plasmids (100 ng), and 10 ng of pRL-TK (as an internal control). Twenty hours after transfection, cells were collected and performed with luciferase assays. Values are presented as the mean ± SD from three independent experiments.

To further determine the structure domains of FMDV 3A that responsible for the interactions with RIG-I, MDA5 and VISA, a series of truncated mutants of FMDV 3A, RIG-I, MDA5, and VISA were generated (Fig. 4a–c). The results suggested that the N-terminal 51 amino acids of 3A was required for its interaction with RIG-I, MDA5, and VISA (Fig. 4d–f). We next examined which domains of RIG-I/MDA5/VISA are required for its interaction with FMDV 3A. The results also showed that CARD and helicase domains of RIG-I/MDA5, and C-terminal amino acids (360–540) of VISA interacted with FMDV 3A (Fig. 4g,h). In reporter assays, it was found that the full-length and N-terminal 102 aminol acids of FMDV 3A inhibited SeV-, RIG-I-, MDA5-, and VISA-triggered IFN-β and ISRE activations (Fig. 5a,b), while C-terminal 51 aminol acids (103–153) of the protein had minor effects (Fig. 5c,d). These results suggest that the N-terminal 51 amino acids of FMDV 3A interacted with the VISA/RIG-I/MDA5 and the N-terminal 102 amino acids of FMDV 3A inhibited the IFN-β signaling pathway.

### FMDV 3A inhibits the expression of the RIG-I, MDA5, and VISA

**Figure 6:**
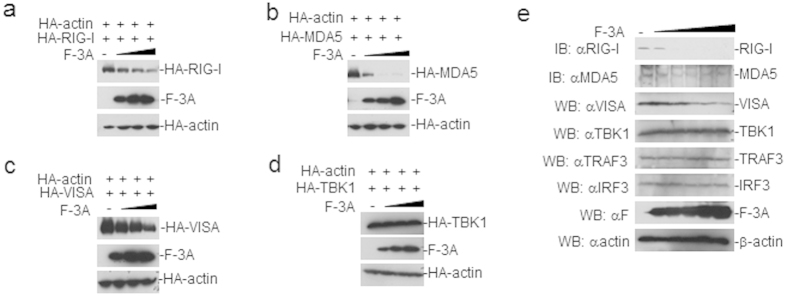
FMDV 3A inhibits the expression of the RIG-I, MDA5 and VISA. (**a–d**) The effects of FMDV 3A on the expression of the RIG-I, MDA5 and VISA. HEK293T (1 × 10^5^) cells were co-transfected with increased amounts of pCAGGS-Flag-3A (F-3A) (0, 0.5, 1.0, and 2 μg) and decreased amounts of pCAGGS (2.0, 1, 1.5, and 0 μg) and indicated plasmids expressing pRK-HA-RIG-I, pRK-HA-MDA5, pRK-HA-VISA or pRK-HA-TBK1 (0.2 μg). As a control, pRK-HA-actin (0.015 μg) was transfected with above plasmids. At 48 hour post transfection, the cell lysate was analyzed by Western blotting using mouse anti-Flag and mouse anti-HA monoclonal antibodies. HA-actin was used as a loading control. (**e**) Dose-dependent effects of FMDV 3A on the expression of endogenous RIG-I, MDA5, VISA, TBK1, TRAF3, and IRF3. HEK293T cells (1.6 × 10^7^) were co-transfected with increased amounts of pCAGGS-Flag-3A (F-3A) (0, 4, 8.0, 12, 16, 24 and 32 μg) and decreased amounts of pCAGGS (32, 28, 24, 20, 16, 8 and 0 μg) for 24 h. A small volume of the protein lysate was collected to detected VISA, TBK1, TRAF3, and IRF3 by western blotting. Other protein lysate was used for immunoprecipitation with protein G-beads coupled to polyclonal antibodies directed against RIG-I or MDA5. The immunoprecipitation was analyzed by immunoblots with the polyclonal antibodies against RIG-I and MDA5.

After confirming the interactions of 3A with RIG-I, MDA-5, and VISA, we further investigate the possible the effects of FMDV 3A on the expression of RIG-I, MDA5, and VISA. Co-transfection of FMDV 3A with RIG-I, MDA5, and VISA in HEK293T cells led to reduced levels of RIG-I, MDA5, and VISA (Fig. 6a–c), but not TBK1 (Fig. 6d). To determine if 3A inhibited the expression of endogenous RIG-I, MDA5, and VISA, HEK293T cells were transfected with FMDV 3A. As shown in Fig. 6e, the expression of endogenous RIG-I, MDA5, and VISA were inhibited, but TBK1, TRAF3, and IRF3 levels were unaffected. Collectively, these results suggest that FMDV 3A inhibits the expressions of the RIG-I, MDA5 and VISA proteins.

### FMDV 3A inhibits the expression of the RIG-I, MDA5, and VISA by disrupting their mRNA level

**Figure 7:**
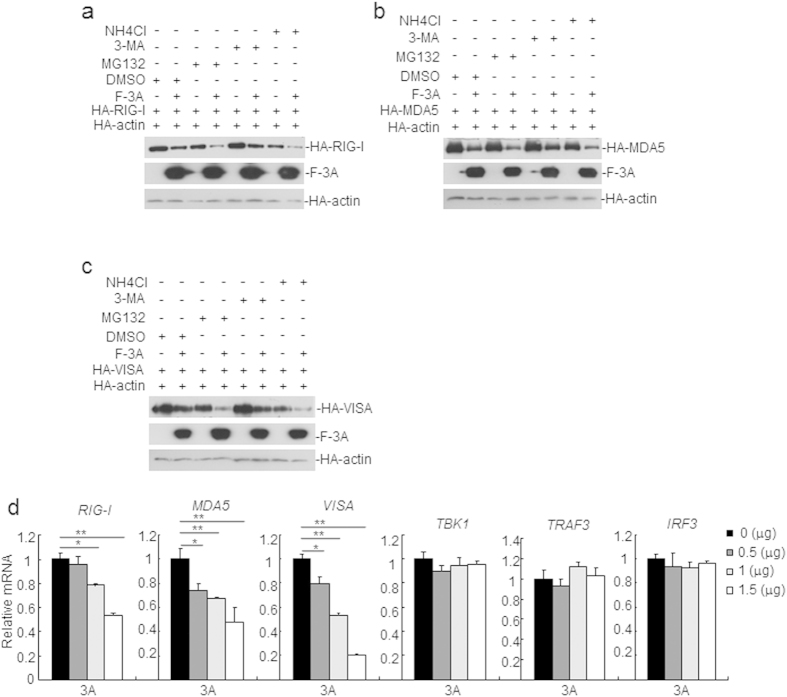
FMDV 3A inhibits the expression of the RIG-I, MDA5, and VISA by disrupting their mRNA level. (**a–c**) FMDV 3A inhibits the expressions of the RIG-I, MDA5, and VISA not at translational level. HEK293T cells were transfected with FMDV 3A or empty vector (1.0 μg), pRK-HA-actin (0.015 μg) and the indicated expression plasmids (0.2 μg). As a control, pRK-HA-actin (0.015 μg) was also transfected with above plasmids. At 18 h post-transfection, cells were treated with dimethyl sulfoxide (DMSO), MG-132 (20 μM), 3-MA (0.5 mg/ml), or NH_4_Cl (20 mM) for 6 h and subjected to western blotting analysis. (**d**) FMDV 3A inhibits the mRNA abundances of RIG-I, MDA5, and VISA. HEK293T cells were transfected with 3A or an empty vector (EV) for 24 h. Total RNA was extracted form cells and RT-PCR was performed to test the expression levels of *Rig-i*, *Mda5*, *Visa*, *Tbk1*, *Traf3*, *Irf3*, and *Gapdh*. Results were expressed as the relative fold change in mRNA levels compared with those in cells transfected with EV. The expression levels were normalized to GAPDH. Values are presented as the mean ± SD from three independent experiments.

The above results showed that FMDV 3A inhibits the expressions of the RIG-I, MDA5 and VISA. It is intriguing to find out if FMDV 3A inhibits the expressions of RIG-I, MDA5 and VISA at translational levels. Treatment with a proteasome inhibitor MG-132, autophagy inhibitor 3-MA, and lysosome inhibitor NH4Cl were not significantly blocked the protein reduction (Fig. 7a–c). In real time PCR experiments, it was found that FMDV 3A inhibited the mRNA abundance of RIG-I, MDA5 and VISA, but not TBK1, TRAF3 and IRF3 (Fig. 7d). These data demonstrate that that FMDV 3A reduced the expression of the RIG-I, MDA5 and VISA by inhibiting their mRNA level, not at translational levels.

## Discussion

Activation of the innate immune system in mammals upon in response to viral infections, leads to the expression of hundreds of antiviral genes that control the spread of infections^23^. The IFN signaling pathway is a crucial component of the innate antiviral response.

FMDV is a member of the *Aphthovirus* genus within the *Picornaviridae* family. Its pathophysiology is likely related to its ability to evade the innate immune system of the host. Some studies have shown that FMDV proteins inhibit the IFN pathway, and therefore evade host innate immunity^18^,^21^,^24^. The mechanisms that FMDV use to manipulate the host for its replication and to evade the host immune response, are not fully understood. Although there are known FMDV-mediated inhibitory mechanisms that affect the initiation and effector phases of the innate immune response, little is known about the effects of FMDV on the signaling transductions. This pathway involves RIG-I-, MDA5-, and VISA-mediated type I IFN production, and is regulated by a sophisticated interplay between host and viral proteins. In the current study, we revealed that the expressions of RIG-I, MDA5, and VISA are reduced by FMDV 3A, which results in inhibition of type I IFN production. The findings could help to explain the influence of FMDV on RIG-I- and MDA-5 signaling transduction pathways, and further improve our understandings of the mechanisms by which FMDV evades the host innate immune system of the host.

RIG-I and MDA-5 play important roles in innate immunity. Recently, several viruses have been reported to use various strategies to disrupt the functions of RIG-I and MDA-5. The influenza virus NS1 protein can antagonize the RIG-1-VISA pathway by interacting with RIG-1 and VISA^25^. The V proteins of the *Paramyxoviruses*, including simian virus 5, human parainfluenza virus 2, mumps virus, SeV, and Hendra virus, were shown to disrupt the MDA5-IFN-β pathway via direct interactions with MDA5^26^,^27^. Other studies showed that several viruses disrupt the function of VISA to evade host innate immunity. The NS3-4A protease of hepatitis C virus inhibits the IFN signaling pathway by cleaving the C-terminal end of VISA to disrupt its interaction with the outer mitochondrial membrane^28^,^29^,^30^. The HBx protein of hepatitis B virus binds with VISA and promotes its degradation to inhibit IFN-β production^31^,^32^. Viruses of the *Picornaviridae* family disrupt the function of VISA through various mechanisms. The 3ABC protein of Hepatitis A virus co-localizes with VISA at the mitochondria and degrades VISA^33^. The rhinovirus 2A^pro^ and 3C^pro^ proteases, and 3C^pro^ of coxsackievirus B3 inhibit the IFN signaling pathway by cleaving VISA^34^. The 2A^pro^ of enterovirus 71 inhibits type I IFN production by cleaving VISA at Gly209, Gly251, and Gly265^35^. All of the above studies were focused on the degradations of the proteins of the IFN signaling pathway by virus-encoded proteases. Therefore, our finding that FMDV 3A inhibited the expression of the RIG-I, MDA5, and VISA provides a new insight into how non-protease FMDV-derived proteins can affect the IFN signaling pathway. FMDV 3A has no sequence homology to any other known proteases. We postulated that other proteins mediates the inhibition of the expressions of RIG-I, MDA5, and VISA proteins by FMDV 3A.

Many viruses have developed mechanisms to subvert the IFN pathway. The nonstructural proteins of bovine respiratory syncytial virus (BRSV) block the induction of genes encoding IFN-α/β, which results in the increased virulence of BRSV^36^. The replication and virulence of West Nile virus is enhanced via resistance to IFN-α/β^37^. The PB2 subunit of influenza virus RNA polymerase inhibits the expression of IFN-β, thereby affecting its virulence^38^. Previous studies have suggested that there is a correlation between IFN expression and the virulence of a virus, but some studies have focused on FMDV pathogenicity and virulence. FMDV 3A consists of 153 amino acids that are partially conserved. The first half of the 3A coding region, which is highly conserved among all FMDVs, encodes an N-terminal hydrophilic domain and a hydrophobic domain capable of binding to membranes^39^. The 3A protein is multifunctional and plays important roles in virus replication, virulence, and determination of host-range^40^,^41^. Previous studies have shown that deletion of amino acids 93–102 of 3A protein in a Taiwanese strain (O/TAW/97) of FMDV is associated with an inability to cause disease in cows. Further research suggested that this deletion does not affect FMDV replication efficiency in BHK-21 (hamster), PK-15 (porcine), and FPK (fetal porcine) cell lines. However, virus replication efficiency was reduced in the FBK (fetal bovine) cell line, although this alone cannot account for the inability of FMDV to replicate in bovine cells. Another Asian strain of FMDV with a second deletion within 3A (amino acids 133–143) or a series of substitutions, revealed that changes in the genome in addition to the original deletion are responsible for the porcinophilic properties of current Asian viruses in this lineage^39^. In FMDV, deletions of 19 to 20 amino acids between 3A residues 87–106 results in reduced virulence in cattle^42^. A recombinant O1 Campos virus harboring a 20 amino acid deletion in 3A exhibited reduced virulence in cattle^43^. It remain unclear that there is a relationship between the amino acid composition of 3A and the IFN signaling pathway, which affects the virulence of FMDV. We demonstrated that amino acids 1–102 of FMDV 3A inhibits the IFN-β signaling pathway, which provides new information regarding the influence of IFN signaling on FMDV virulence. Further studies are required to determine whether the roles of the IFN response reported here culminate in FMDV pathophysiology and which key amino acids of FMDV 3A inhibit the IFN-β signaling pathway.

In summary, FMDV 3A adversely affects RIG-I, MDA5, and VISA-mediated type I IFN production by inhibiting the mRNA levels of these genes. Furthermore, we have identified a key domain (amino acids 1–102) of FMDV 3A that inhibits the IFN-β signaling pathway. Taken together, the findings reveal a novel mechanism of FMDV 3A-mediated evasion of host innate immunity.

## Methods

### Cells, viruses and reagents

PK-15 (porcine kidney cells) and HEK293T cells were cultured in Dulbecco’s modified Eagle’s medium (DMEM) supplemented with 10% fetal bovine serum (FBS). We used mouse monoclonal antibodies against Flag, β-actin (Sigma, St. Louis, MO, USA), hemagglutinin (HA) (Covance), Myc, green fluorescent protein (GFP), and TBK1 (Santa Cruz Biotechnology, Santa Cruz, CA, USA). Rabbit polyclonal antibodies against IRF3 and phosphorylated IRF3 were also employed (Santa Cruz Biotechnology, Santa Cruz, CA, USA). IFN-γ (PeproTech, Rocky Hill, NJ, USA) was used for IFN stimulation. Gamma Bind G Plus-Sepharose was purchased from Amersham Biosciences and M-MLV reverse transcriptase was from Invitrogen. The Sendai virus (SeV) used in the experiments and rabbit antibodies against VISA, MDA5, TRAF3, and RIG-I were previously described^44^,^45^,^46^. The type O FMDV was propagated in PK-15 cells, and the supernatants of infected cells were clarified and stored at −80 °C.

### Plasmids

The generation of luciferase reporter plasmids encoding the promoters for ISRE, IRF1, and IFN-β, along with mammalian expression plasmids for RIG-I (CARD), RIG-I (helicase), RIG-I, MDA5, VISA, TBK1, TRAF3, TRAF6, IRF3, IKKα, IKKβ, P65 and VISA mutants have been described previously^7^,^44^,^46^,^47^,^48^,^49^. The genes of FMDV proteins were amplified from cDNA of the type O FMDV, strain Tibet/CHA/99 (GenBank accession no. AJ539138). FMDV proteins cDNA were cloned into the pCAGGS vector using EcoRI and XhoI restriction enzymes. A series of FMDV 3A mutants were cloned into the pRK-GFP or pMSCV vectors using EcoRI and XbaI restriction enzymes generated from pCAGGS-3A by conventional PCR techniques with the mutagenesis primers listed in Table 1. All plasmids were verified by sequencing.

### Transfection and reporter assays

HEK293T cells (5 × 10^4^) in 48-well plates were transfected with 100 ng of reporter plasmid, 10 ng of pRL-TK (Promega) (as an internal control), and 100 ng indicate plasmids. At 24 hours post transfection, the cells were treated with SeV (moi: 1.0) or IFNγ (100 ng/ml) or left untreated for 12 h, and the whole-cell extracts were prepared for the analysis of dual-luciferase activities. The activities of the reporter genes, including firefly luciferase and renilla luciferase, were determined using a dual-luciferase reporter 1000 assay system (Promega) according to the manufacturer’s instructions. The data represented the firefly luciferase activity normalized to the renilla luciferase activity. Three independent experiments were carried out in duplicate.

### Real-time RT-PCR

The expression of IFN-β, CXCL10, ISG56 and RANTES were examined by a relative quantitative real-time RT-PCR. HEK293T cells were transfected with the FMDV 3A or empty vector (4 μg) for 24 h. The cells were treated with SeV (moi: 1.0) or left untreated for 12 h. The total RNA was extracted from the HEK293T cells with TRIzol (catalog no. 15596026; Invitrogen) and treated with DNase I to remove potential genomic DNA contaminants. The isolated RNA was then reverse transcribed to cDNA with Moloney murine leukemia virus reverse transcriptase according to the manufacturer’s instructions. The transcriptional level of mRNA was quantified by real time RT-PCR with SYBR Permix Ex Taq II (catalog no. DRR081A; TaKaRa) in a LightCycler 480 II real-time PCR system (Roche). The target gene expression was normalized to the expression of a reference gene, the glyceraldehyde-3-phosphate dehydrogenase (GAPDH) gene. The primers for genes are listed in Table 1. Relative fold changes in gene expression were determined by the threshold cycle (2^−ΔΔCt^) method.

The viral genome copies in FMDV-infected PK-15 cells were quantified by a quantitative real-time RT-PCR. Total RNA of the FMDV-infected cells was extracted using TRIzol as described above. Quantification of genome copies of FMDV was performed as described previously^50^.

### Co-immunoprecipitation and western immunoblotting

Transfected HEK293T cells from 10-cm dishes were lysed in l ml of lysis buffer (20 mM Tris-HCl pH 7.4–7.5, 150 mM NaCl, 1 mM EDTA, 1% Nonidet P-40, 10 μg/mL aprotinin, 10 μg/mL leupeptin, and 1 mM phenylmethylsulfonyl fluoride). For each sample, 0.8 mL of cell lysate was incubated with 0.5 μg of appropriate antibody and 25 μl of 50% (v/v) slurry of protein G agarose beads (Amersham Biosciences) at 4 °C for 2 h. Agarose beads were washed three times with 1 mL of lysis buffer containing 500 mM NaCl. Precipitates were subjected to sodium dodecyl sulfate polyacrylamide gel electrophoresis and then western immunoblotting analysis.

### Virus infection and treatment

PK-15 cells were transfected with plasmids for 12 h then treated with puromycin (1 mg/ml) for 24 h followed by infection with the type O FMDV (MOI: 0.1). After 2 h, the viral inoculum was removed and the infected cells were washed twice with phosphate-buffered saline (PBS) (pH 7.4) and re-fed with DMEM containing 2% FBS. IFN-β mRNA abundance or FMDV genome copies in PK-15 cells were assessed using a relative quantitative RT-PCR assay or using a quantitative RT-PCR assay. To examine effect of FMDV 3A on the expressions of RIG-I, MDA5 and VISA, HEK293T cells co-transfected with RIG-I, MDA5 or VISA and 3A or empty vector for 18 h followed by treatment with DMSO, MG132 (20 μM), 3-MA (0.5 mg/ml), NH4Cl (20 mM) for 6 h. The samples were harvested and analyzed using western immunoblotting.

### Statistical analysis

Statistical analysis was performed using the SPSS17.0 software. The student's t test was used for a comparison of three independent treatments. For the test, a P value < 0.05 was considered significant (^*^) while P value < 0.01, very significant (^**^).

## Additional Information

**How to cite this article**: Li, D. *et al.* Foot-and-mouth disease virus non-structural protein 3A inhibits the interferon-β signaling pathway. *Sci. Rep.* **6**, 21888; doi: 10.1038/srep21888 (2016).

## Figures and Tables

**Table 1:** Primers used in this study.

Primers	Sequences (5′-3′)	Use
2B-Flag-F	TTCACGTCTCGAATTCGCCACCATGCCCTTCTTCTTCTCCGACGT	Amplification of 2B
2B-Flag-R	TTCACGTCTCCTCGAGTCACTTGTCATCATCGTCCTTATAGTCCTGTTTCTCTGCTCTCTCA	
2C-Flag-F	TTCACGTCTCGAATTCGCCACCATGCTCAAAGCACGTGACATCAA	Amplification of 2C
2C-Flag-R	TTCACGTCTCCTCGAGTCACTTGTCATCATCGTCCTTATAGTCCTGCTTGAAGATCGGGTGG	
3A-Flag-F	TTCACGTCTCGAATTCGCCACCATGATCTCAATTCCTTCCCAAA	Amplification of 3A
3A-Flag-R	TTCACGTCTCCTCGAGTCACTTGTCATCATCGTCCTTATAGTCTTCAGCTTGTGGTTGCTCCT	
3B-Flag-F	TTCAGGTCTCGAATTCGCCACCATGGGACCCTACGCCGGACCA	Amplification of 3B
3B-Flag-R	TTCAGGTCTCCTCGAGTCACTTGTCATCATCGTCCTTATAGTCCTCAGTGACAATCAGGTTCT	
3C-Flag-F	CCGGAATTCGCCACCATGAGTGGTGCCCCACCGACC	Amplification of 3C
3C-Flag-R	CCGACTCGAGTCACTTGTCATCATCGTCCTTATAGTCCTCGTGATGTGGTTCGGGGT	
3D-Flag-F	CCGGAATTCGCCACCATGGGGTTGATTGTGGACACCAG	Amplification of 3D
3D-Flag-R	CCGACTCGAGTCACTTGTCATCATCGTCCTTATAGTCTGCGTCACCACACACGGCGT	
3A-GFP-F	CGGAATTCATGATCTCAATTCCTTCCCAAAAATC	Amplification of 3A
3A-GFP-R	GCTCTAGATTCAGCTTGTGGTTGCTCC	
3A(1–51)-GFP-F	CGGAATTCATGATCTCAATTCCTTCCCAAAAATC	Amplification of 3A(aa 1–51)
3A(1–51)-GFP-R	GCTCTAGAAGCGCGTTTCACAAATGAAG	
3A(52–102)-GFP-F	CGGAATTCATGTTCAAGCGCCTGAAGGAAAATTTTG	Amplification of 3A(aa 52–102)
3A(52–102)-GFP-R	GCTCTAGAATCTGTGGTGATGTTTGCTTTC	
3A(103–153)-GFP-F	CGGAATTCATGGACAAGACTCTTGACGAGGC	Amplification of 3A(aa 103–153)
3A(103–153)-GFP-R	GCTCTAGATTCAGCTTGTGGTTGCTCC	
hIFN-β-F	TTGTTGAGAACCTCCTGGCT	Q-PCR for detection of human *ifnb* gene
hIFN-β-R	TGACTATGGTCCAGGCACAG	
hCXCL10-F	GGTGAGAAGAGATGTCTGAATCC	Q-PCR for detection of human *cxcl10* gene
hCXCL10-R	GTCCATCCTTGGAAGCACTGCA	
hISG56-F	GCCTTGCTGAAGTGTGGAGGAA	Q-PCR for detection of human *isg56* gene
hISG56-R	ATCCAGGCGATAGGCAGAGATC	
hRANTES-F	GGCAGCCCTCGCTGTCATCC	Q-PCR for detection of human *rantes* gene
hRANTES-R	GCAGCAGGGTGTGGTGTCCG	
hRIG-I-F	CACCTCAGTTGCTGATGAAGGC	Q-PCR for detection of human *rig-i* gene
hRIG-I-R	GTCAGAAGGAAGCACTTGCTACC	
hMDA5-F	GCTGAAGTAGGAGTCAAAGCCC	Q-PCR for detection of human *mda5* gene
hMDA5-R	CCACTGTGGTAGCGATAAGCAG	
hVISA-F	ATGGTGCTCACCAAGGTGTCTG	Q-PCR for detection of human *visa* gene
hVISA-R	TCTCAGAGCTGCTGTCTAGCCA	
hTBK1-F	CAACCTGGAAGCGGCAGAGTTA	Q-PCR for detection of human *tbk1* gene
hTBK1-R	ACCTGGAGATAATCTGCTGTCGA	
hTRAF3-F	ACAAGTGCAGCGTCCAGACTCT	Q-PCR for detection of human *traf3* gene
hTRAF3-R	GCCTTGATCTGCTGGTTTGTCC	
hIRF3-F	TCTGCCCTCAACCGCAAAGAAG	Q-PCR for detection of human *irf3* gene
hIRF3-R	TACTGCCTCCACCATTGGTGTC	
hGAPDH-F	GAGTCAACGGATTTGGTCGT	Q-PCR for detection of human *gapdh* gene
hGAPDH-R	GACAAGCTTCCCGTTCTCAG	
pIFN-β-F	GGCTGGAATGAAACCGTCAT	Q-PCR for detection of procine *ifnb* gene
pIFN-β-R	TCCAGGATTGTCTCCAGGTCA	
pGAPDP-F	ACATGGCCTCCAAGGAGTAAGA	Q-PCR for detection of procine *gapdh* gene
pGAPDP-R	GATCGAGTTGGGGCTGTGACT	
